# Association between pace of aging estimated from blood DNA methylation and all-cause mortality: the HUNT study

**DOI:** 10.1186/s13148-026-02070-8

**Published:** 2026-05-20

**Authors:** Yi-Qian Sun, Ilona Urbarova, Lin Jiang, Therese Haugdahl Nøst, Xiao-Mei Mai

**Affiliations:** 1 Department of Clinical and Molecular Medicine, Norwegian University of Science and Technology (NTNU), Trondheim, Norway; 2 Department of Pathology, Clinic of Laboratory Medicine, St. Olavs Hospital, Trondheim, Norway; 3 Center for Oral Health Services and Research Mid-Norway (TkMidt), Trondheim, Norway; 4 Department of Community Medicine, Faculty of Health Sciences, UiT The Arctic University of Norway, Tromsø, Norway; 5 Department of Public Health and Nursing, Norwegian University of Science and Technology, Trondheim, Norway; 6 Clinic of Cardiology, St. Olav’s Hospital, Trondheim, Norway; 7 HUNT Research Centre, Norwegian University of Science and Technology, Levanger, Norway; 8 Levanger Hospital, Nord-Trøndelag Hospital Trust, Levanger, Norway; 9 HUNT Centre for Molecular and Clinical Epidemiology, Norwegian University of Science and Technology, Trondheim, Norway

**Keywords:** Biological age, DNA methylation, Epigenetic clocks, HUNT, Mortality, Pace of aging

## Abstract

**Background:**

Epigenetic clocks, developed using blood DNA methylation data, can be used to estimate biological ages and pace of aging. We aimed to identify potential determinants of the pace of aging, estimated using blood DNA methylation, and to investigate the association between the pace of aging and all-cause mortality in a population-based Norwegian cohort with repeated DNA methylation measurements.

**Methods:**

This study included 140 cancer-free controls from a lung cancer nested case-control study within the Trøndelag Health Study (HUNT). DNA methylation was measured in blood samples in both HUNT2 (1995–97) and HUNT3 (2006–08), 11 years apart. The pace of aging was estimated using two established measures of biological age, DNAmPhenoAge and DNAmGrimAge2, and two direct measures of pace of aging, DunedinPoAm and DunedinPACE, all derived from blood DNA methylation data. All-cause mortality was followed up until 2023.

**Results:**

There was a moderate to excellent reliability of the repeated measurements, with intraclass correlation coefficient (ICC) values ranged from 0.69–0.91. University education appeared to be associated with a slower pace of aging, while smoking and obesity were associated with a faster pace. A one-standard deviation increase in the pace of aging, as measured by DNAmGrimAge2, was associated with a hazard ratio (HR) of 2.42 (95% confidence interval (CI) 1.25 to 4.68) for all-cause mortality in HUNT2, and a HR of 2.30 (95% CI 1.22 to 4.33) in HUNT3 after adjustment for the established risk factors.

**Conclusions:**

The pace of aging, as estimated using blood DNA methylation, appears to be an independent predictor of all-cause mortality. This measure may reflect the combined influence of genetic, lifestyle, and environmental factors on individual aging trajectories.

**Supplementary Information:**

The online version contains supplementary material available at 10.1186/s13148-026-02070-8.

## Introduction

Epigenetic clocks developed using blood DNA methylation data may provide insightful information on chronological and biological ages and causality for aging-related traits [1, 2]. The predicted chronological age was derived from an elastic net regression model that used numerous cytosine-phosphate-guanine dinucleotide (CpG) sites of DNA methylation, with actual chronological age as the outcome [3–5]. Different numbers of CpG sites, ranging from 71–514, were ultimately included in the final linear predictors for chronological age [3–5]. The predicted biological age was derived using a similar method and principle, with the outcome of the regression model defined by multiple clinical biomarkers or time-to-death [6, 7]. Further, the predicted biological age can be used to estimate pace of aging, defined as the residuals from regressing predicted biological age on reported chronological age [8, 9]. Pace of aging conceptionally represents an individual rate of decline across multiple organ systems and exhibits significant variations among individuals who are at the same chronological age [10]. Additionally, Belsky et al. used changes in approximately 20 biomarkers over 12 or 20 years as outcomes and developed algorithms to directly predict the pace of aging from a single DNA methylation measurement using 46 or 173 CpG sites, respectively [8, 10].

Evaluation of biological age and pace of aging in independent cohorts with data on multimorbidity and mortality, particularly in cohorts with repeated DNA methylation measurements, is crucial for establishing the applicability of such epigenetic clocks in assessing their associations with health outcomes. However, the independent associations between DNA methylation-based pace of aging and multimorbidity, as well as mortality remain underexplored [9, 11, 12]. In this study, we aimed to study the pace of aging, as estimated from four established epigenetic clocks, in relation to sociodemographic and lifestyle variables and risk of all-cause mortality. Additionally, we investigated change in the pace of aging per year between two time points using repeated DNA methylation measurements, representing how rapidly the pace of aging changed over time, in relation to all-cause mortality.

## Methods

### Study design and population

This study included 140 cancer-free controls from a lung cancer nested case-control study within the Trøndelag Health Study (HUNT Study) [13], based on the availability of repeated DNA methylation measurements. The initial study comprised 140 incident lung cancer cases diagnosed between 2009 and 2013 and 140 age- (± 3 years) and sex-matched controls [14].

Briefly, genome-wide DNA methylation was measured in blood samples collected in HUNT2 (1995–1997) and HUNT3 (2006–2008) [14, 15]. About 500 ng of DNA was isolated from peripheral blood cells and bisulphite-converted using EZ DNA methylation kit (Zymo Research, CA, USA). Methylation levels at over 850,000 sites were quantified using the Infinium MethylationEPIC BeadChip kit v1 (Illumina Inc., CA, USA) [14]. After quality control and functional normalization, DNA methylation estimates were presented as beta-values ranging from 0–1. Data on age, sex, education, body mass index (BMI, kg/m^2^), serum 25-hydroxyvitamin D [25(OH)D] levels, and lifestyle factors, such as smoking status with pack-years, alcohol consumption, physical activity, and multimorbidity, were collected and measured in HUNT2 and HUNT3. Multimorbidity included a range of self-reported severe diseases such as myocardial infarction, angina pectoris, stroke, diabetes, and cancer. A variable of severe diseases was defined as the presence of any of these conditions. These variables were classified in detail in previous HUNT studies [16–18]. The HUNT Research Centre receives information about deaths from all causes of HUNT participants from the Norwegian National Population Registry. All-cause mortality was followed up until June 15, 2023.

### Estimated chronological and biological age, as well as the pace of aging

The seven epigenetic clocks used in the current study are summarized in Supplementary Table 1. First, we calculated three DNA methylation-based chronological ages, i.e. DNAmAge, DNAmAgeHannum, and Zhang’s clock, to validate the concept of the epigenetic clock in HUNT2 [3–5]. Second, we calculated the biological age in HUNT2 using DNA methylation data based on two established measures: DNAmPhenoAge and DNAmGrimAge2 [6, 7]. In brief, the intercepts (if applicable) and coefficients for each CpG site used in the prediction formula for the three chronological clocks and DNAmPhenoAge were obtained from the original studies [3–6], while the coefficients for DNAmGrimAge2 [7] were downloaded from Biolearn [19]. The estimated chronological and biological ages were obtained using the formulas provided in the respective studies, for example:

$$ \begin{gathered} Predicted{\text{ }}age{\text{ }} \hfill \\ \quad \quad \quad = {\text{ }}b_{0} + {\text{ }}b_{1} *CpG1{\text{ }} + {\text{ }} \cdot \cdot \cdot {\text{ }} + {\text{ }}b_{m} *CpG_{m} \hfill \\ \end{gathered} $$

Here, b0 represents the intercept, b1 is the coefficient for CpG1, and bm is the coefficient for CpGm where m denotes the total number of CpG sites in the study. CpG1 and CpGm refer to beta-values in HUNT. The final predicted age may require specific transformations based on the individual studies [3, 7].

Third, the age acceleration was estimated as the residuals from regressing the estimated biological age, DNAmPhenoAge and DNAmGrimAge2, on reported chronological age [8, 9]. In addition, two measures of the pace of aging derived from blood DNA methylation data, DunedinPoAm and DunedinPACE, were directly calculated using the R packages *DunedinPoAm38* and *DunedinPACE*, respectively [8, 10]. As both age acceleration and pace of aging refer to measures of the aging rate, we use only the term pace of aging throughout the manuscript for simplicity. In total, four measures of the pace of aging were included in the current study: two measures derived from DNAmPhenoAge and DNAmGrimAge2, as well as two direct measures DunedinPoAm and DunedinPACE.

Pace of aging was treated as both a continuous and a categorical variable, and the latter was classified into three groups such as slow [one standard deviation (SD) or more below the mean], average (within one SD of the mean), and fast (one SD or more above the mean). All calculations were repeated using DNA methylation data from HUNT3. The purposes of using methylation measurements from HUNT3 were (1) to validate findings from HUNT2; (2) to assess the reliability of DNA methylation as markers for the pace of aging over time; and (3) to estimate the longitudinal change in the pace of aging between HUNT2 and HUNT3.

### **Relationships between sociodemographic and lifestyle variables and pace of aging**,** as well as pace of aging in relation to all-cause mortality**

We investigated the individual relationship of education, smoking status, alcohol consumption, physical activity, BMI, season-standardized serum 25(OH)D levels [18], and severe diseases with the pace of aging. Additionally, we examined whether the pace of aging was independently associated with all-cause mortality. The analyses were conducted using DNA methylation data from HUNT2 and replicated using data from HUNT3. 

### Change in the pace of aging per year based on repeated measurements in relation to all-cause mortality

Lastly, we evaluated change in the pace of aging per year between HUNT2 and HUNT3 in relation to all-cause mortality. The change in the pace of aging per year was defined as (pace of aging in HUNT3–pace of aging in HUNT2) divided by the number of follow-up years between these two time points, representing how rapidly the pace of aging increased or decreased over time.

### Statistical analysis

All statistical analyses and plots were conducted and generated using R (version 4.4.0). Pearson correlation coefficients were calculated to assess the strength of linear relationship between two sets of values, whereas root mean squared error (RMSE) was calculated to measure the average difference between predicted and actual values. We calculated the intraclass correlation coefficient (ICC) to evaluate the absolute agreement of the pace of aging measures between the two time points (HUNT2 and HUNT3). ICC is a widely used reliability index in test-retest analysis. It reflects both the degree of correlation and agreement between repeated measurements. The ICC and its 95% confidence intervals (CIs) were derived using a two-way mixed-effects model based on the mean of the two sets of estimates [20].

To identify possible determinants of the pace of aging, linear regression models were used with pace of aging as the outcome, adjusting for potential confounders. When education was the exposure, chronological age and sex were adjusted for. When smoking status, alcohol consumption, physical activity, and BMI was exposure respectively, chronological age, sex and education were adjusted for in addition to the other three variables due to their possible bidirectional relationships. When serum 25(OH)D levels and severe diseases were the exposure respectively, chronological age, sex, education, smoking status, alcohol consumption, physical activity, and BMI were adjusted for in addition to severe diseases/serum 25(OH)D for each other.

Cox proportional hazards regression models, with chronological age as the time scale, were used to estimate hazard ratios (HRs) and 95% CIs for all-cause mortality in relation to the pace of aging or change in the pace of aging per year. The proportional hazards assumption was tested and confirmed for all variables included in the model. The main model was adjusted for important confounders such as sex, education, smoking with pack-years, and BMI to minimize over-fitting of the model. When change in the pace of aging per year being the exposure, we further adjusted for the baseline pace of aging in HUNT2 to assess its independent association with all-cause mortality.

To compare different measures, the pace of aging and the change in the pace of aging per year were standardized as z-scores when their relationships with various variables as possible determinants and hazard of all-cause mortality were examined, respectively.

## Results

Among the 140 participants, DNA methylation in the blood was measured in 137 in HUNT2 and 135 in HUNT3, and 133 participants had DNA methylation measured at both time points. Table 1 presents the characteristics of the 137 and 135 participants, respectively. The mean age in HUNT2 was 55.6 years (SD 8.4), and 45.3% were women. Approximately 50% of the participants were ever smokers (former or current). More than half of the participants were overweight or obese in HUNT2, with a mean BMI of 25.7 kg/m^2^ (SD 3.1). From HUNT2 to HUNT3, the proportion of participants with physical inactivity or low activity decreased, while the proportions of those with frequent alcohol consumption, obesity and severe diseases increased.

**Table 1:** Characteristics of the controls in a nested case-control design of the HUNT study

Characteristics	HUNT2 (1995–1997)	HUNT3 (2006–2008)
No. of participants (n)	137	135
Age (years)	55.6 ± 8.4	66.8 ± 8.5
Sex (women/men)	45.3/54.7	46.7/53.3
Education [primary school (reference)/high school/university or higher/unknown^*^]	39.4/31.4/26.3/2.9	40.0/31.9/25.2/3.0
Smoking status [never (reference)/former/current/unknown^*^]	48.2/27.7/21.9/2.2	44.4/36.3/17.8/1.5
Alcohol consumption [never (reference)/1–4 times per month/≥5 times per month/unknown^*^]	33.6/48.9/13.9/3.6	7.4/69.6/20.7/2.2
Physical activity [inactive (reference)/low/moderate/high/unknown^*^]	19.0/17.5/29.2/8.0/26.3	8.9/8.9/31.1/8.1/43.0
BMI (kg/m^2^)	25.7 ± 3.1	26.5 ± 3.5
BMI [kg/m^2^: <25.0 (reference)/25.0–29.9 (overweight)/≥30.0 (obese)]	42.3/49.6/8.0	33.3/53.3/13.3
Season-standardized 25(OH)D nmol/L	51.5 ± 15.2	50.8 ± 15.0
Season-standardized 25(OH)D [nmol/L: <30.0/30.0–49.9/50.0–74.9 (reference)/≥75.0]	7.3/38.0/49.6/5.1	7.4/40.7/45.9/5.9
Severe diseases [no (reference)/yes]	97.8/2.2	91.1/8.9

25(OH)D: 25-hydroxyvitamin D; BMI: body mass index; HUNT: Trøndelag Health Study

Data are presented as mean ± standard deviation (continuous variables) or percentage of participants (categorical variables)

^*^unknown: represents the category with missing information

**Fig. 1:**
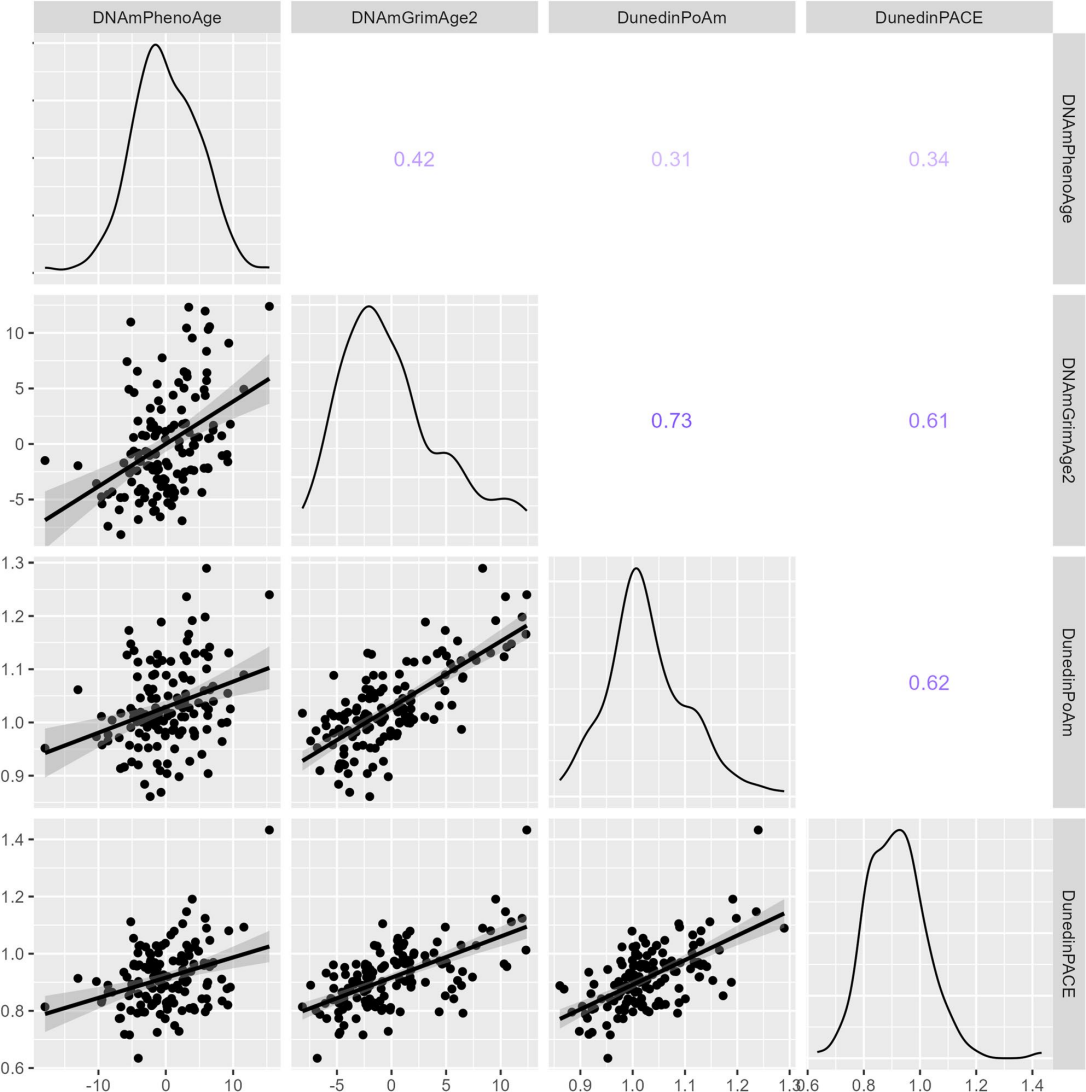
The correlations between the four measures of the pace of aging in HUNT2 (n = 137). The numbers in the upper-right part of the figure represent Pearson correlation coefficients, with larger values and darker colors indicating stronger correlations. The diagonal histograms display the distribution of individual measures of the pace of aging, while the bottom-left part of the figure shows pairwise correlations among the four measures of the pace of aging. The x-axis and y-axis strip labels represent the values of the measures in the histograms and scatter plots. HUNT: Trøndelag Health Study

The correlation coefficients between predicted and reported chronological ages ranged from 0.88–0.96 for the three epigenetic clocks in HUNT2, indicating a high degree of correlation (Supplementary Table 2). RMSE was from 4.3 to 11.9 for the three clocks in HUNT2. Similar results were shown using the DNA methylation data from HUNT3. The measures of the pace of aging, based on DNAmPhenoAge and DNAmGrimAge2, had means of 0 and ranged from − 17.9 to 15.4 and − 8.2 to 12.4, respectively. Negative values indicate a slower pace of aging, while positive values indicate a faster pace of aging. The other measures of the pace of aging, DunedinPoAm and DunedinPACE, represent the ratio of biological to chronological aging per year, ranging from 0.63–1.43 in HUNT2. The correlation coefficients across the four measures of the pace of aging in HUNT2 ranged from 0.31–0.73, with DNAmGrimAge2 and DunedinPoAm showing the strongest correlation (Fig. 1). The correlation coefficients based on DNA methylation data from HUNT3 ranged from 0.31 to 0.69 and showed similar patterns to those observed in HUNT2 (Supplementary Fig. 1). The ICC values for the four measures of the pace of aging, derived from the DNA methylation data in both HUNT2 and HUNT3, ranged from 0.69 for DunedinPoAm to 0.91 for DNAmGrimAge2, indicating moderate to excellent reliability across repeated measurements (Supplementary Table 3) [20].

**Fig. 2:**
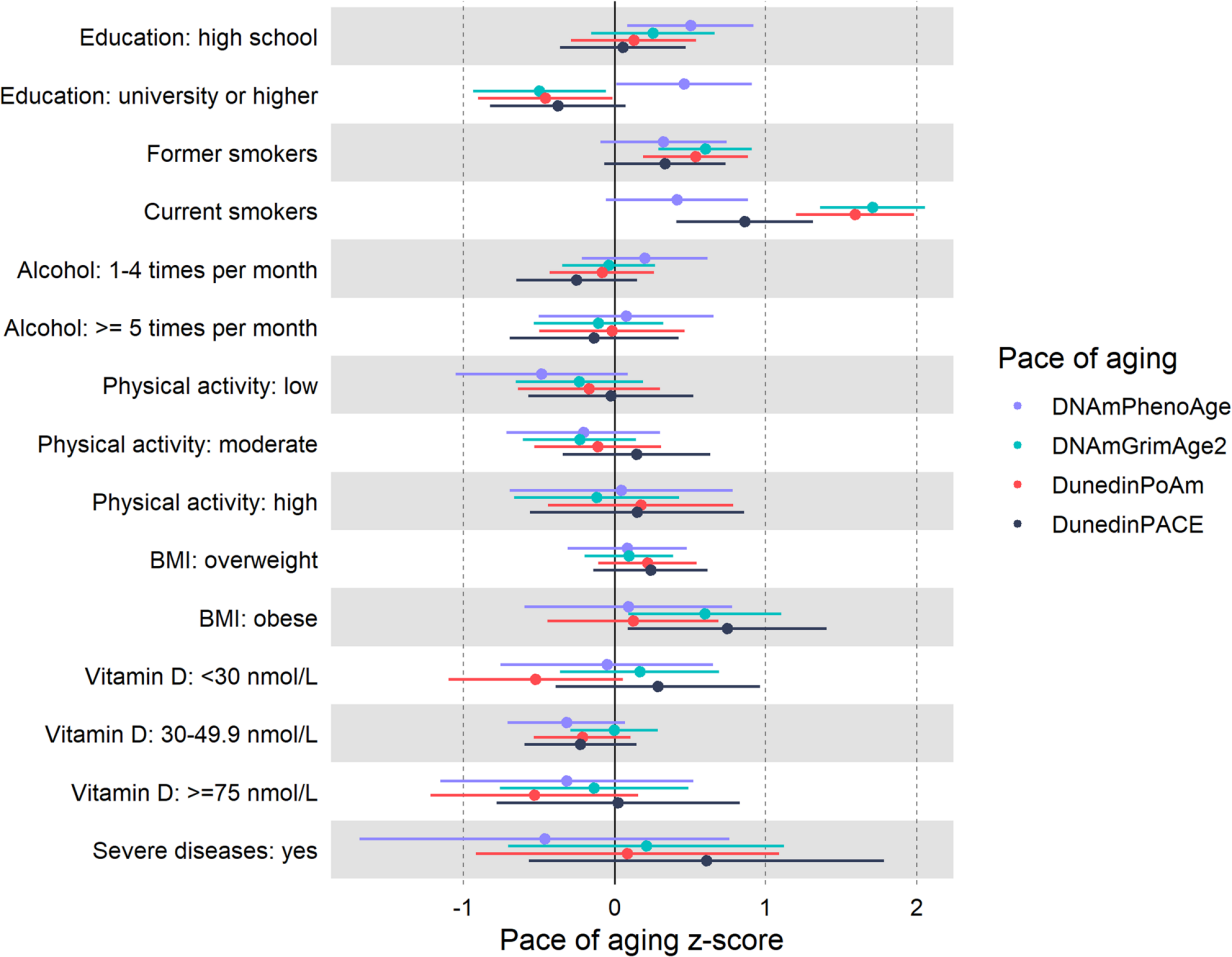
The associations between sociodemographic and lifestyle variables and pace of aging in HUNT2 (n = 137). The z-score of the pace of aging was used to compare different measures. The reference groups of exposures are indicated in Table 1. Education as exposure: chronological age and sex were adjusted. Smoking status, alcohol consumption, physical activity, and BMI as exposure, respectively: chronological age, sex, and education were adjusted for in addition to the other three variables. Serum 25(OH)D and severe diseases as exposure, respectively: chronological age, sex, education, smoking status, alcohol consumption, physical activity, and BMI were adjusted for in addition to severe diseases/serum 25(OH)D for each other. 25(OH)D: 25-hydroxyvitamin D; BMI: body mass index; HUNT: Trøndelag Health Study

Relationships between sociodemographic and lifestyle variables and the pace of aging were examined using the reference groups indicated in Table 1. As illustrated in Fig. 2, university education appeared to be associated with a slower pace of aging, whereas former and current smoking were associated with a faster pace of aging in HUNT2. These associations were consistent among the measures of DNAmGrimAge2, DunedinPoAm, and DunedinPACE. In addition, individuals who were current smokers showed a faster pace of aging than former smokers with the same pack-years for the mentioned three measures (Supplementary Fig. 2). Obesity was associated with a faster pace of aging using the measures DNAmGrimAge2 and DunedinPACE in HUNT2 and HUNT3 (Fig. 2, Supplementary Fig. 3). Other factors such as alcohol consumption, physical activity, serum 25(OH)D levels, and severe diseases did not appear to be associated with the pace of aging.

During a median follow-up of 26.6 years, 32 out of 137 participants in HUNT2 died (11 women and 21 men). In the main model, a one-SD increase in the pace of aging was associated with a 46 to 142% higher likelihood of all-cause mortality across the four measures with wider 95% CIs (Table 2). Significant associations were observed for DNAmGrimAge2 (HR 2.42, 95% CI 1.25 to 4.68) and DunedinPACE (HR 1.99, 95% CI 1.09 to 3.64). The survival probabilities of the three categorical groups, i.e. slow, average, and fast, were generally distinguishable on the Kaplan-Meier curves for DNAmGrimAge2 and DunedinPACE although the confidence interval areas overlapped (Supplementary Fig. 4). We replicated the above analyses using the DNA methylation data from HUNT3 with a median follow-up of 15.4 years. The results were generally consistent with those from HUNT2, with significant association observed for DNAmGrimAge2 in HUNT3 (HR 2.30, 95% CI 1.22 to 4.33, Table 2, Supplementary Fig. 5B). The strongest association with all-cause mortality was observed for DNAmGrimAge2 consistently in HUNT2 and HUNT3 (Table 2).

**Table 2:** Associations between the Pace of aging in HUNT2 or HUNT3 and all-cause mortality

Pace of aging	Crude model^*^		Main model^†^	
	HR	95% CI	HR	95% CI
*HUNT2 (n = 137)*				
DNAmPhenoAge	1.79	1.13–2.84	1.46	0.87–2.43
DNAmGrimAge2	1.89	1.34–2.66	2.42	1.25–4.68
DunedinPoAm	1.78	1.17–2.72	1.64	0.88–3.04
DunedinPACE	2.26	1.40–3.64	1.99	1.09–3.64
*HUNT3 (n = 135)*				
DNAmPhenoAge	1.54	1.03–2.29	1.19	0.77–1.85
DNAmGrimAge2	2.39	1.61–3.56	2.30	1.22–4.33
DunedinPoAm	1.63	1.11–2.39	1.67	0.97–2.87
DunedinPACE	1.37	0.89–2.12	1.15	0.70–1.89

HR: hazard ratio; HUNT: Trøndelag Health Study

HR was calculated using Cox proportional hazards regression with age as the time scale, in relation to one z-score increase in the pace of aging

^*^Crude model: chronological age as the time scale

^†^Main model: chronological age as the time scale, adjusted for sex, education, smoking with pack-years, and body mass index (as continuous) in HUNT2 or HUNT3

As shown in Table 3, one SD increase in the change of the pace of aging per year (an accelerated pace of aging) from HUNT2 to HUNT3 was associated with a significantly increased hazard of all-cause mortality with DNAmGrimAge2 in the adjusted models (HR 1.76, 95% CI 1.13 to 2.74), even after accounting for the baseline pace of aging in HUNT2.

**Table 3:** Associations between the change in the Pace of aging per year from HUNT2 to HUNT3 and all-cause mortality (n = 133)

Pace of aging	Crude model^*^		Model 1^†^		Model 2^ǂ^	
	HR	95% CI	HR	95% CI	HR	95% CI
DNAmPhenoAge	1.02	0.71–1.47	1.02	0.67–1.56	1.09	0.70–1.71
DNAmGrimAge2	1.23	0.83–1.81	1.50	1.00–2.25	1.76	1.13–2.74
DunedinPoAm	1.08	0.72–1.60	1.28	0.81–2.04	1.62	0.92–2.84
DunedinPACE	0.74	0.49–1.11	0.90	0.58–1.40	0.99	0.63–1.55

HR: hazard ratio; HUNT: Trøndelag Health Study

HR was calculated using Cox proportional hazards regression with age as the time scale, in relation to one z-score increase in the change of pace of aging per year

^*^Crude model: chronological age as the time scale

^†^Model 1: chronological age as the time scale, adjusted for sex, education, smoking with pack-years, and body mass index (as continuous) in HUNT3

^ǂ^Model 2: adjusted for baseline pace of aging in HUNT2 in addition to Model 1

## Discussion

### Main findings

We evaluated the measures of pace of aging derived from four established epigenetic clocks, i.e. DNAmPhenoAge, DNAmGrimAge2, DunedinPoAm, and DunedinPACE using repeated DNA methylation measurements, in the Norwegian HUNT Study. Among these, the measures of DNAmGrimAge2 and DunedinPoAm demonstrated the strongest correlation. Additionally, these four measures of the pace of aging showed moderate to excellent reliability across the repeated measurements between HUNT2 and HUNT3. University education appeared to be associated with a slower pace of aging, whereas smoking and obesity were associated with a faster pace of aging. An increase in the pace of aging, measured by DNAmGrimAge2, had significant and strongest association with an increased hazard of all-cause mortality in both HUNT2 and HUNT3. In addition, an accelerated pace of aging (an increase in the change of the pace of aging per year) from HUNT2 to HUNT3, measured by DNAmGrimAge2, was significantly associated with a higher likelihood of all-cause mortality.

### Comparison with previous studies

The ICC values for the four measures of pace of aging in the current study indicated moderate to excellent reliability across repeated measurements. Belsky et al. used repeated DNA methylation measurements to assess whether participants’ pace of aging changed over time in two studies [8, 10]. Both studies demonstrated exceptional test-retest reliability, with ICC values of 0.89 and 0.96, respectively, which were somewhat higher than the ICC values in the current study. However, their ICC values should be considered as internal validation [8, 10].

Our results support the latest findings that social disadvantages across the life course, as indicated by low educational attainment, may accelerate aging and increase risk of 66 age-related diseases [21]. Moreover, Faul et al. evaluated the pace of aging measures, including DNAmPhenoAge and DunedinPACE, in relation to education and various lifestyle factors [11]. They found that low educational attainment and smoking were associated with faster aging, which is consistent with our findings. They also found that obesity and heavy alcohol consumption were linked to faster aging, which aligns with the findings for obesity in the current study but not for alcohol consumption. A recent randomized controlled trial showed that supplementation with 2000 IU of vitamin D per day, combined with an omega-3 supplement for three years, slowed biological aging as measured by DNAmPhenoAge [22]. However, vitamin D supplementation alone or a home exercise program alone did not show significant benefit across any of the four biological age clocks studied, including the three clocks used in the current study, which aligned with our findings. A fast pace of aging was also associated with multimorbidity in previous studies [8, 11], which was not found in the current study. This is likely because our study population was healthier at baseline, as they were initially selected as controls for lung cancer cases and included relatively few individuals with self-reported severe diseases. In general, we emphasize that the imprecise estimates of potential determinants should be considered exploratory, owing to the small sample size and multiple covariate adjustment.

Our crude model, which used reported chronological age as the time scale, demonstrated that an increase in the pace of aging was associated with all-cause mortality. The magnitudes of associations for the four measures were somewhat larger and less precise than those reported by Ying et al., who adjusted for chronological age and sex [12]. The more precise estimates observed in this previous study are not surprising, given the study had a larger sample size (*n* = 1488). Our main model showed a similar association with all-cause mortality per SD increase in the pace of aging, as measured by DNAmPhenoAge, compared with a Swedish study that adjusted for similar covariates (HRs 1.46, 1.19, and 1.23 in HUNT2, HUNT3, and the Swedish study, respectively) [23]. In addition, our main model showed a 99% higher likelihood of all-cause mortality per SD increase in the pace of aging in HUNT2, as measured by DunedinPACE, compared with a 23% higher risk reported in a Finnish study [9] after adjustment for similar covariates. This discrepancy could be due to a shorter follow-up period in the Finnish study, as we observed a 15% higher risk in HUNT3 [9]. The survival curves in the study by Belsky et al. showed a clearer separation between the slow, average, and fast pace of aging categorical groups compared to those in the current study [8]. This is likely due to the larger sample size and limited adjustment for covariates including only sex and chronological age in the referred study.

A previous study used repeated blood DNA methylation measurements to examine their relationships with frailty [24]. However, no previous study has examined the potential effect of change in the pace of aging over time on all-cause mortality. Using repeated DNA methylation measurements in HUNT2 and HUNT3, we found that change in the pace of aging per year measured by DNAmGrimAge2 was independently associated with all-cause mortality, even after adjustment for the baseline pace of aging. However, changes in the pace of aging over time for the other three measures showed no association, highlighting the need for further research on the impact of longitudinal changes in the pace of aging.

### Potential mechanisms and causal insights

Correlations among the four measures of pace of aging range from weak to moderate, suggesting that they capture partially distinct biological processes. Therefore, they should not be considered interchangeable.

The epigenetic clocks examined in this study were developed entirely using machine learning. Their underlying molecular mechanisms remain largely unknown [1]. To explore these mechanisms, a computational approach using large and multi-tissue DNA methylation datasets was applied [25]. Levine et al. clustered approximately 5000 clock CpG sites into twelve modules, each with distinct age-related dynamics and tissue patterns. They found that the established epigenetic clocks comprise different proportions of these modules, suggesting they are composites of diverse biological processes. This may explain the discordant behaviors observed across the clocks in the current study. DNAmGrimAge (predecessor of DNAmGrimAge2) and DunedinPoAm shared similar module compositions, which may explain why these two measures had the strongest correlation in the current study. In addition, differences among the four clocks likely reflect how biological age or pace of aging was defined during model development. DNAmGrimAge2 used time-to-death as its outcome, while the others relied on multiple clinical biomarkers, possibly explaining its strongest association with all-cause mortality in the current study.

As no genome-wide association studies of epigenetic clocks for biological age have been conducted, there are currently no genetic instrumental variables available for use in Mendelian randomization analyses to assess the causal relationships between these clocks and health outcomes [26]. Ying et al. identified causal CpG sites for aging traits and built clocks enriched for these sites [2]. However, the clocks examined in the current study, like most other clocks, are not enriched for these causally implicated CpG sites.

### Strengths and limitations

Our study is among the few independent cohorts that have used repeated DNA methylation measurements in blood to evaluate the established epigenetic clocks [23, 27, 28]. There are several advantages of using repeated DNA methylation data. First, a single measurement likely introduces random measurement error [29], therefore, we used the HUNT3 measurements as internal validation of the results derived from HUNT2. Second, by using repeated measurements taken 11 years apart, we were able to assess the reliability of these biomarkers by calculating the ICC values over time. Third, to our knowledge, this is the first study to examine the association between annual change in the pace of aging and all-cause mortality using repeated DNA methylation data. In addition to the repeated measurements, we had access to a comprehensive set of variables, enabling us to study determinants of the pace of aging.

However, our study has several limitations. First, the study sample consisted of approximately 140 middle-aged and older adults from the HUNT study, originally selected as controls for lung cancer cases. Therefore, the generalizability of the findings to the broader Norwegian population or other populations is limited. By saying this, we acknowledge that the statistical inference (i.e. the regression estimates) is not generalizable to populations beyond the one studied. However, the scientific inference, i.e. the evidence of the association between the pace of aging and all-cause mortality in the current study, is broadly generalizable [30, 31]. Second, the sample size was relatively small. This resulted in less precise estimates for some associations and precluded subgroup analyses, such as stratification by sex to explore potential sex-based differences. Third, we lacked data on other biomarkers, including inflammatory cytokines and telomere length, which could have provided further insights into the relationships between biomarkers and the pace of aging. Fourth, there was likely self-reporting bias in certain variables, such as alcohol consumption and physical activity, which may have contributed to the null associations with the pace of aging. Fifth, we examined only four of the many biological age clocks based on DNA methylation [12]. However, these are among the most tested and validated in the literature [1, 12]. Sixth, we calculated the association estimates based on a one-SD increase in the pace of aging to enable comparison between different measures, which resulted in larger estimate sizes. Lastly, although we demonstrated independent association between the pace of aging and all-cause mortality after adjustment for confounders, causal relationship cannot be established due to potential unmeasured confounding and the lack of genetic instrumental variables for epigenetic clocks, as previously discussed.

## Conclusion

Overall, repeated DNA methylation measurements demonstrated moderate to excellent reliability as markers of the pace of aging. The four measures of the pace of aging derived from blood DNA methylation, especially DNAmGrimAge2, were associated with an increased hazard of all-cause mortality in our dataset, independent of the established risk factors. These measures may reflect the combined influence of genetic, lifestyle, and environmental factors on individual aging trajectories.

## Supplementary Information

Below is the link to the electronic supplementary material.

Supplementary Material 1

## Data Availability

Data from the HUNT Study that is used in research projects will, when reasonably requested by others, be made available on request to the HUNT Data Access Committee (hunt@medisin.ntnu.no). The HUNT data access information describes the policy regarding data availability ( [https://www.ntnu.edu/hunt/data](https:/www.ntnu.edu/hunt/data) ).

## Abbreviations

25(OH)D: 25-hydroxyvitamin D
BMI: Body mass index
CI: Confidence interval
CpG: Cytosine-phosphate-guanine dinucleotide
DNA: Deoxyribonucleic acid
HR: Hazard ratio
HUNT Study: The Trøndelag health study
ICC: Intraclass correlation coefficient
RMSE: Root mean squared error
SD: Standard deviation
