# Supplementary material for: Association between pace of aging estimated from blood DNA methylation and all-cause mortality: the HUNT study
Source: Clin Epigenetics. 2026 May 20;18:81. doi: 10.1186/s13148-026-02070-8 (PMC13188620; doi:10.1186/s13148-026-02070-8)
Supplement: Supplementary file 1 — Supplementary Material 1 [file 13148_2026_2070_MOESM1_ESM.docx]

## Supplementary tables

**Supplementary table 1.** Epigenetic clocks used in the current study.

| **Name of epigenetic clock in original paper** | **First author and publication year** | **Tissues and cell types used** | **Method used for the prediction model** | **Illumina array** | **No. of CpG sites studied** | **No. of CpG sites used in prediction model** | **Outcome used in the regression model** |
| --- | --- | --- | --- | --- | --- | --- | --- |
| **Chronological age clocks** | | |  |  |  |  |  |
| DNAmAge (Horvath’s clock) [1] | Horvath 2013 | 51 different tissues and cell types | Elastic net regression | 27K or 450K | 21 369 present on both 27K and 450K | 353 | Chronological age |
| DNAmAgeHannum [2] | Hannum 2013 | Whole blood | Elastic net regression combined with bootstrap approaches | 450K | 450K | 71 | Chronological age |
| Zhang’s clock [3] | Zhang 2019 | Whole blood | Elastic net regression | 450K or EPIC | 319,607 | 514 | Chronological age |
| **Biological age or pace of aging clocks** | | |  |  |  |  |  |
| DNAmPhenoAge [4] | Levine 2018 | Whole blood | Elastic net regression with 10-fold cross-validation | 450K or EPIC | 20 169 present on 27K, 450K, and EPIC | 513 | Biological age (derived from 10 clinical biomarkers) |
| DNAmGrimAge2 [5] | Lu 2022 | Whole blood | Cox regression model with an elastic net penalty | 450K or EPIC | 1030 from DNAmGrimAge | 10 DNAm based biomarkers* + chronological age + sex | Biological age (based on time-to-death) |
| DunedinPoAm [6] | Belsky 2020 | Whole blood | Elastic net regression | 450 or EPIC | All probes on 450 and EPIC | 46 | Pace of aging (based on changes of 18 biomarkers over 12 years) |
| DunedinPACE [7] | Belsky 2022 | Whole blood | Elastic net regression | 450 or EPIC | 20,000 probes (with high ICC) on 450 and EPIC | 173 | Pace of aging (based on changes of 19 biomarkers over 20 years) |

DNAm: DNA methylation

*10 biomarkers using 42 to 211 CpG sites

**Supplementary table 2.** Pearson’s correlation coefficients and RMSE between reported and predicted chronological ages in HUNT2 and HUNT3

| **Predicted chronological age** | **First author and publication year** | **r** | **RMSE** |
| --- | --- | --- | --- |
|  |  | HUNT2 | |
| DNAmAge | Horvath 2013 | 0.88 | 4.3 |
| DNAmAgeHannum | Hannum 2013 | 0.88 | 11.9 |
| Zhang’s clock | Zhang 2019 | 0.96 | 9.2 |
|  |  | HUNT3 | |
| DNAmAge | Horvath 2013 | 0.86 | 4.5 |
| DNAmAgeHannum | Hannum 2013 | 0.88 | 13.5 |
| Zhang’s clock | Zhang 2019 | 0.94 | 5.9 |

HUNT: Trøndelag Health Study; r: correlation coefficient; RMSE: root mean squared error

**Supplementary table 3.** Intraclass correlation coefficient (ICC) of the pace of aging between HUNT2 and HUNT3 (n=133)

| **Pace of aging** | **ICC** | **95% CI** |
| --- | --- | --- |
| DNAmPhenoAge | 0.82 | 0.75 to 0.88 |
| DNAmGrimAge2 | 0.91 | 0.88 to 0.94 |
| DunedinPoAm | 0.69 | 0.49 to 0.80 |
| DunedinPACE | 0.75 | 0.40 to 0.87 |

CI: confidence interval; HUNT: Trøndelag Health Study

## Supplementary figures

**Supplementary figure 1.** The correlations between the four measures of the pace of aging in HUNT3 (n=135)

The numbers in the upper-right part of the figure represent Pearson correlation coefficients, with larger values and darker colors indicating stronger correlations. The diagonal histograms display the distribution of individual measures of the pace of aging, while the bottom-left part of the figure shows pairwise correlations among the four measures of the pace of aging. The x-axis and y-axis strip labels represent the values of the measures in the histograms and scatter plots. HUNT: Trøndelag Health Study.

| 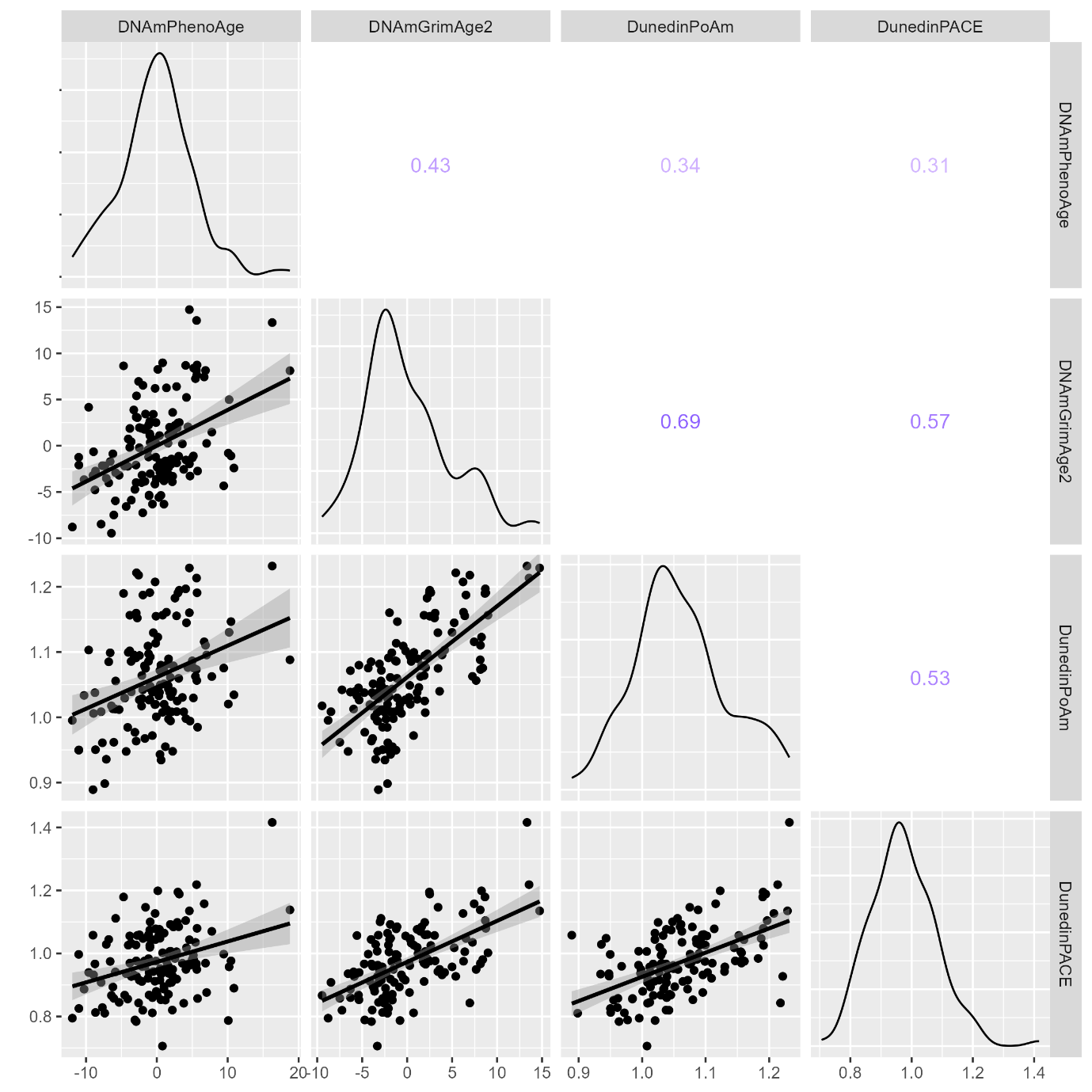 |
| --- |

**Supplementary figure 2.** The associations between smoking status with pack-years and pace of aging in HUNT2 (n=137)

The model was adjusted for chronological age, sex, education, alcohol consumption, physical activity, and body mass index. HUNT: Trøndelag Health Study.

| 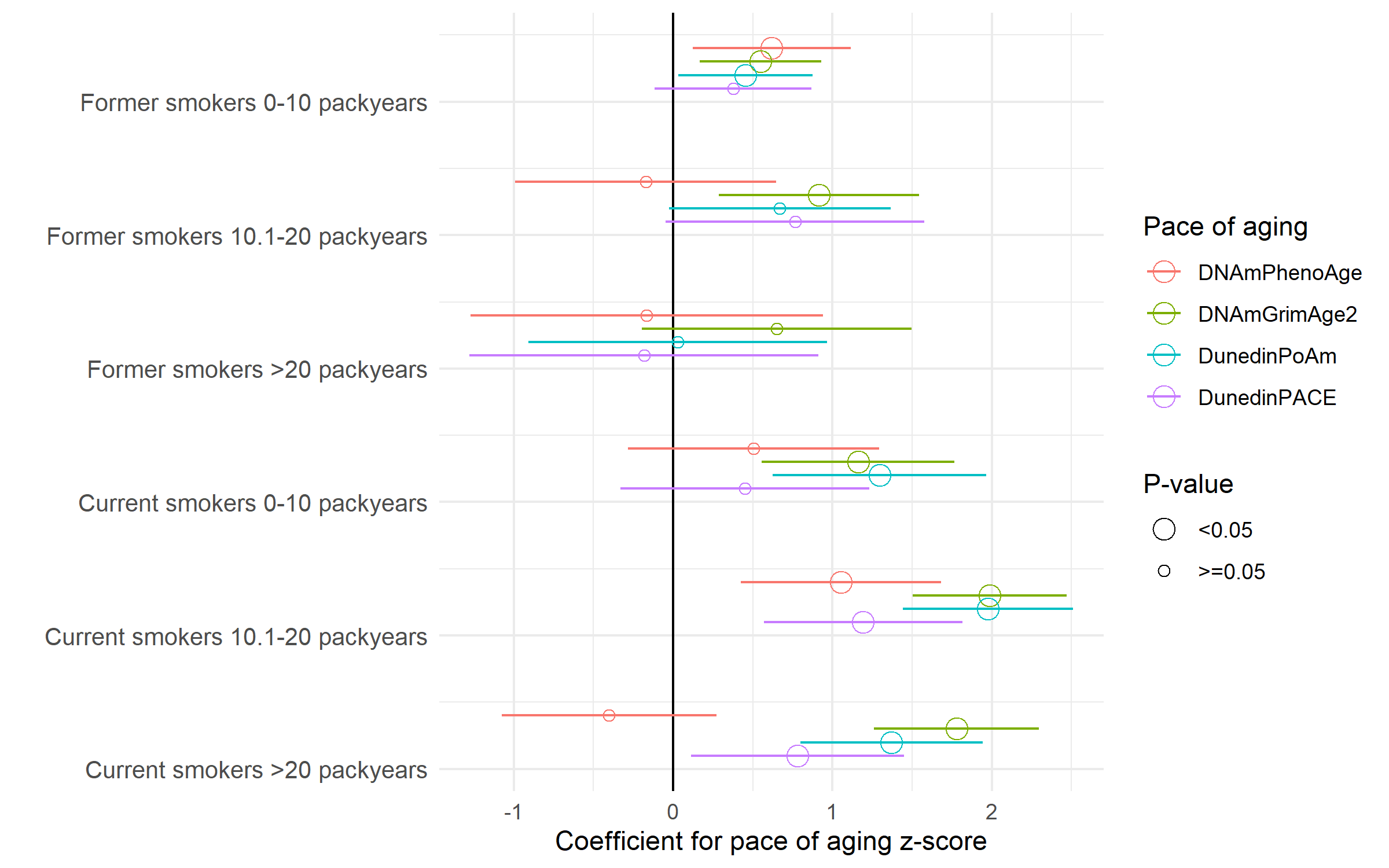 |
| --- |

**Supplementary figure 3.** The associations between sociodemographic and lifestyle variables and pace of aging in HUNT3 (n=135)

The z-score of the pace of aging was used to compare different measures. The reference groups of exposures are indicated in Table 1.

Education as exposure: chronological age and sex were adjusted.

Smoking status, alcohol consumption, physical activity, and BMI as exposure, respectively: chronological age, sex and education were adjusted for in addition to the other three variables.

Serum 25(OH)D and severe diseases as exposure, respectively: chronological age, sex, education, smoking status, alcohol consumption, physical activity, and BMI were adjusted for in addition to severe diseases/serum 25(OH)D for each other.

BMI: body mass index; HUNT: Trøndelag Health Study.

| 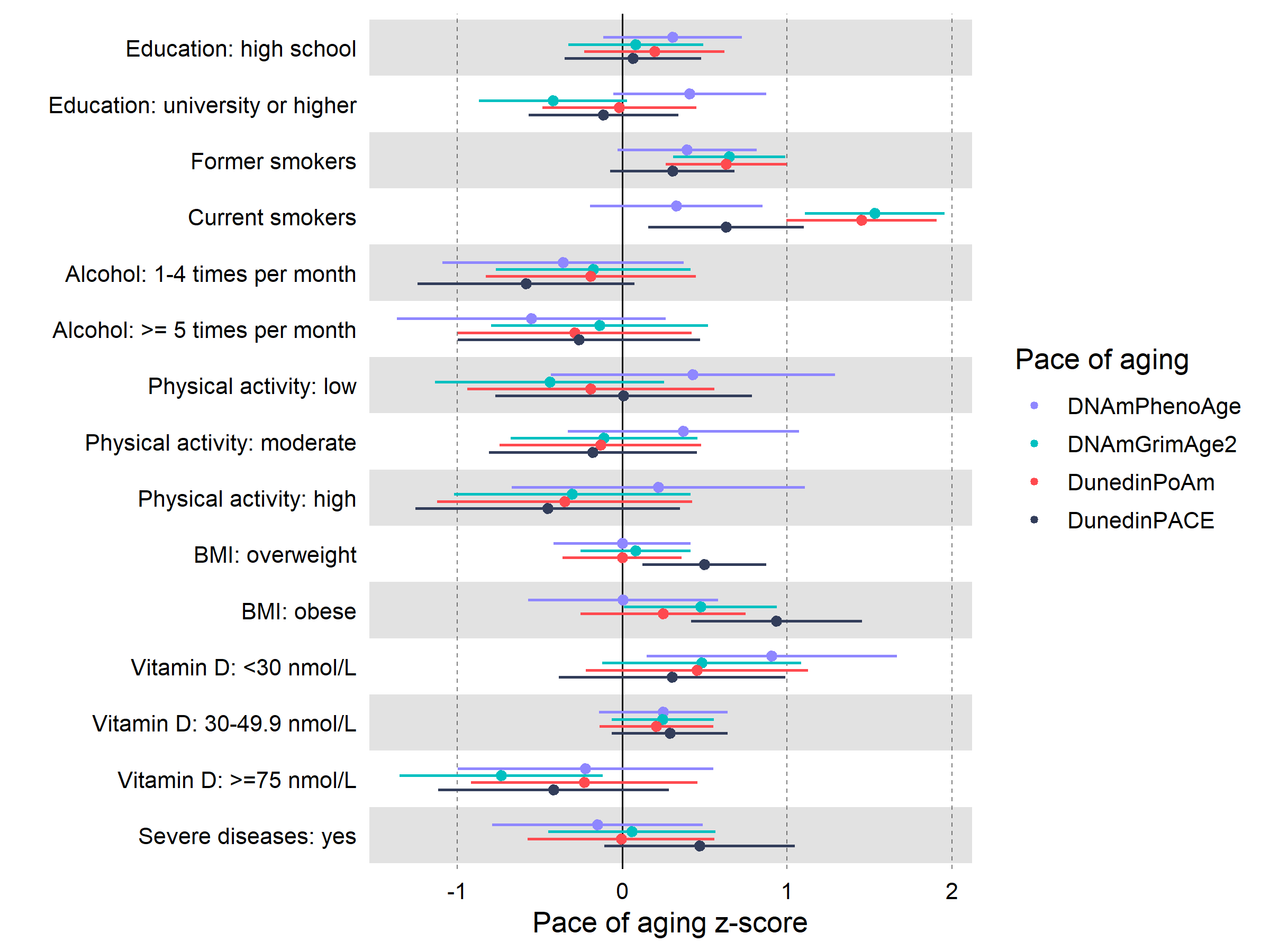 |
| --- |

**Supplementary figure 4.** Kaplan-Meier curves for three categorical groups (slow, average, and fast) of the pace of aging in HUNT2 (n=137)

Slow: Participants with a pace of aging 1 SD or more below the mean; Average: Participants within 1 SD of the mean pace of aging; Fast: Participants with a pace of aging 1 SD or more above the mean.

A) DNAmPhenoAge; B) DNAmGrimAge2; C) DunedinPoAm; D) DunedinPACE. HUNT: Trøndelag Health Study. SD: standard deviation

| A)  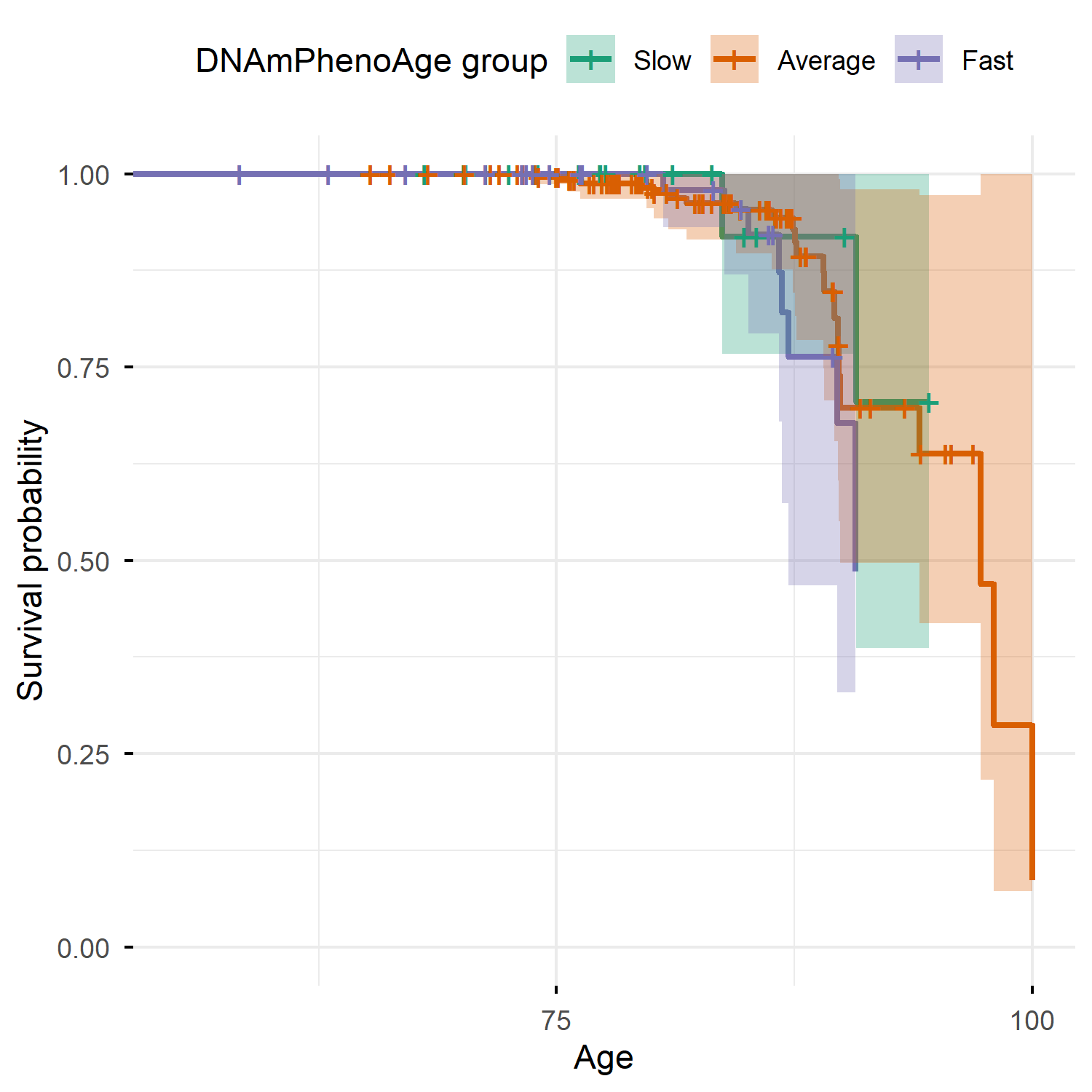 | B)  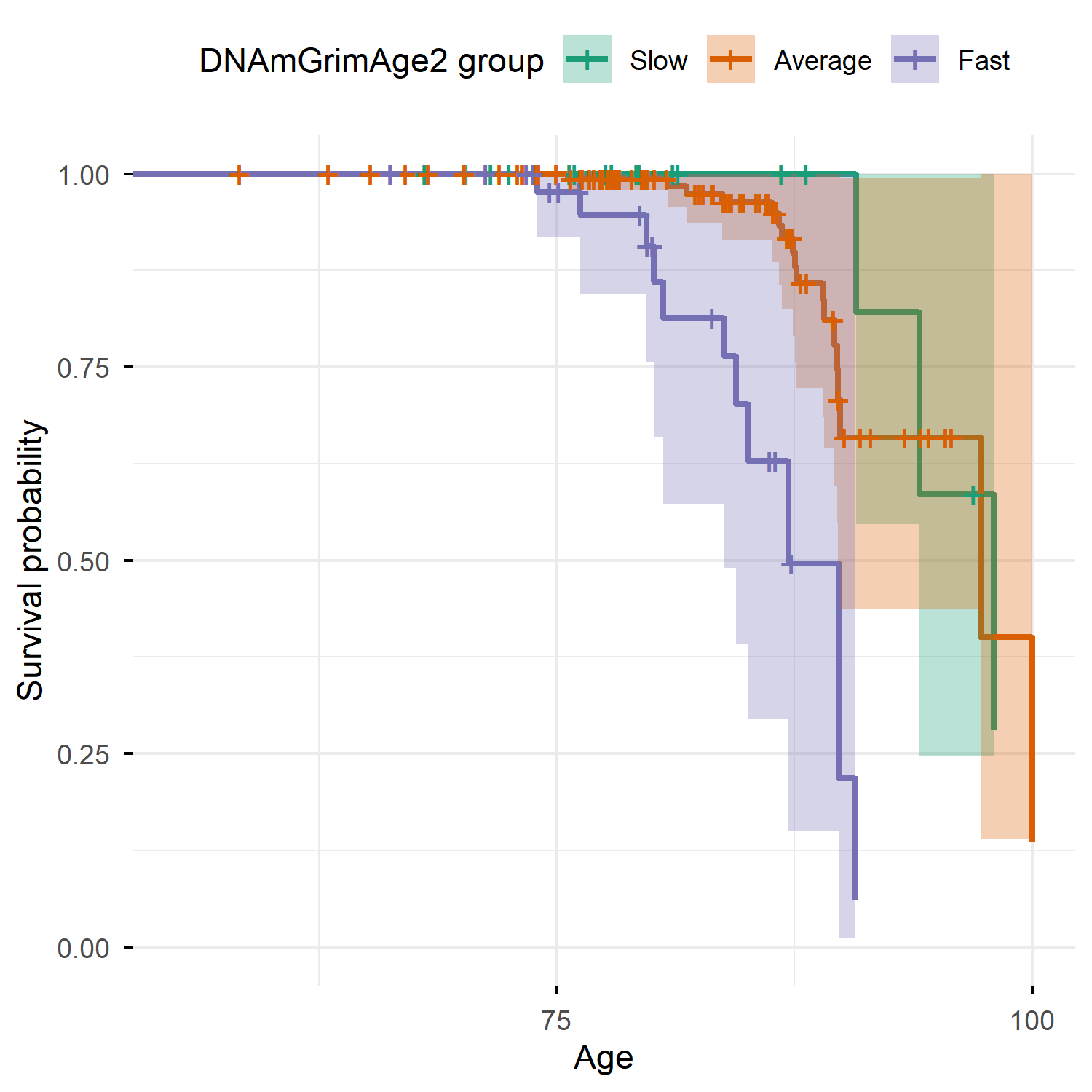 |
| --- | --- |
| C)  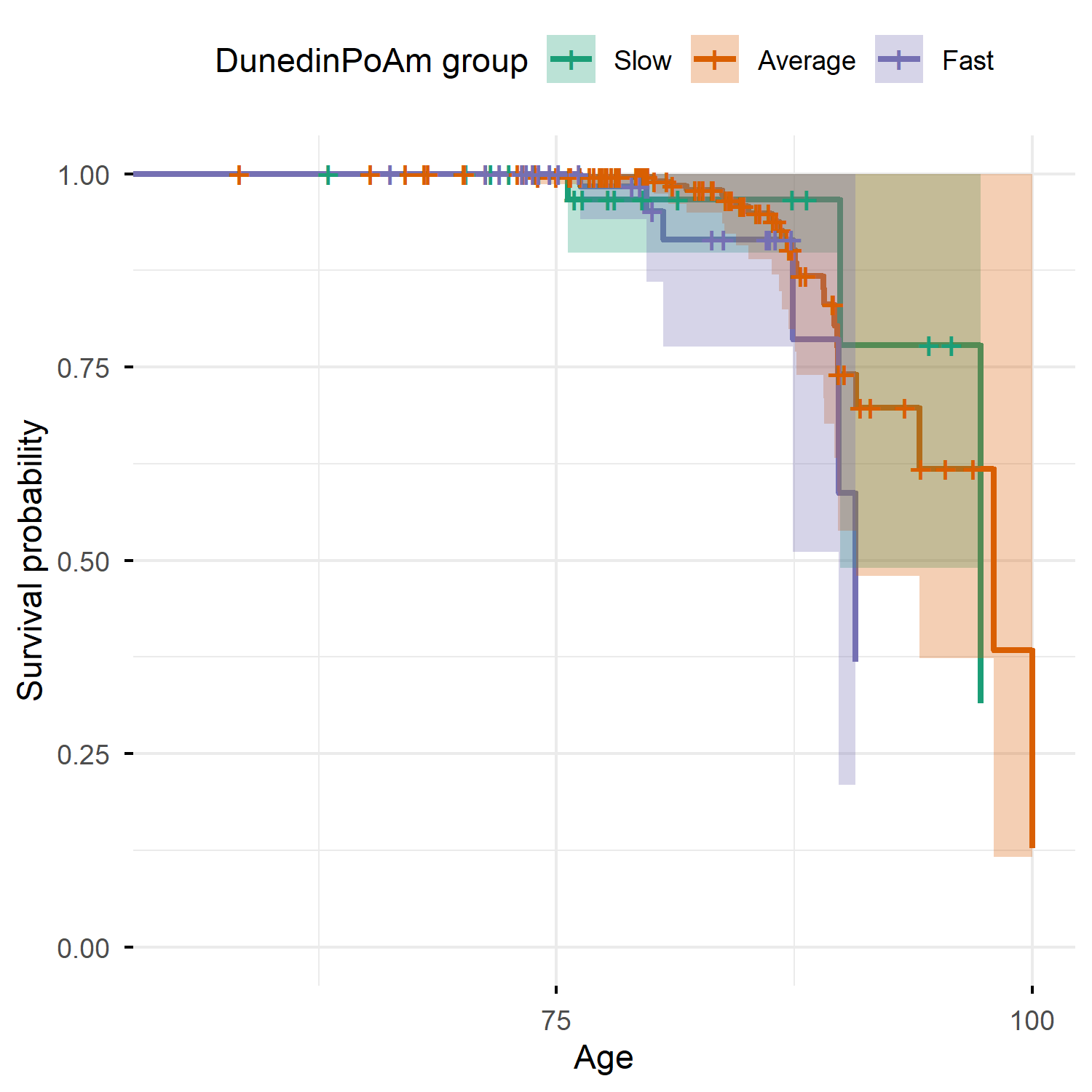 | D)  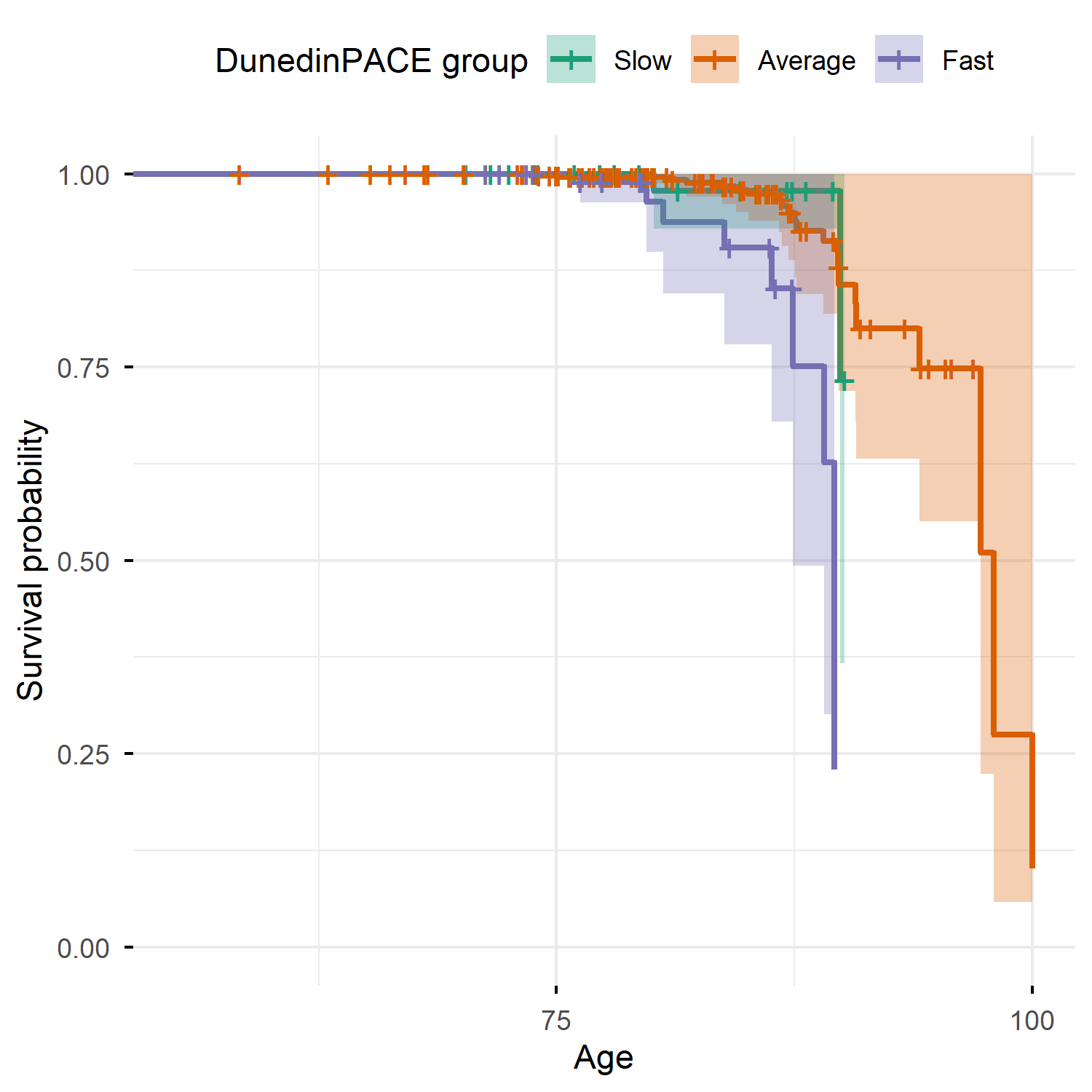 |

**Supplementary figure 5.** Kaplan-Meier curves for three categorical groups (slow, average, and fast) of the pace of aging in HUNT3 (n=135)

Slow: Participants with a pace of aging 1 SD or more below the mean; Average: Participants within 1 SD of the mean pace of aging; Fast: Participants with a pace of aging 1 SD or more above the mean.

A) DNAmPhenoAge; B) DNAmGrimAge2; C) DunedinPoAm; D) DunedinPACE. HUNT: Trøndelag Health Study. SD: standard deviation.

| A)  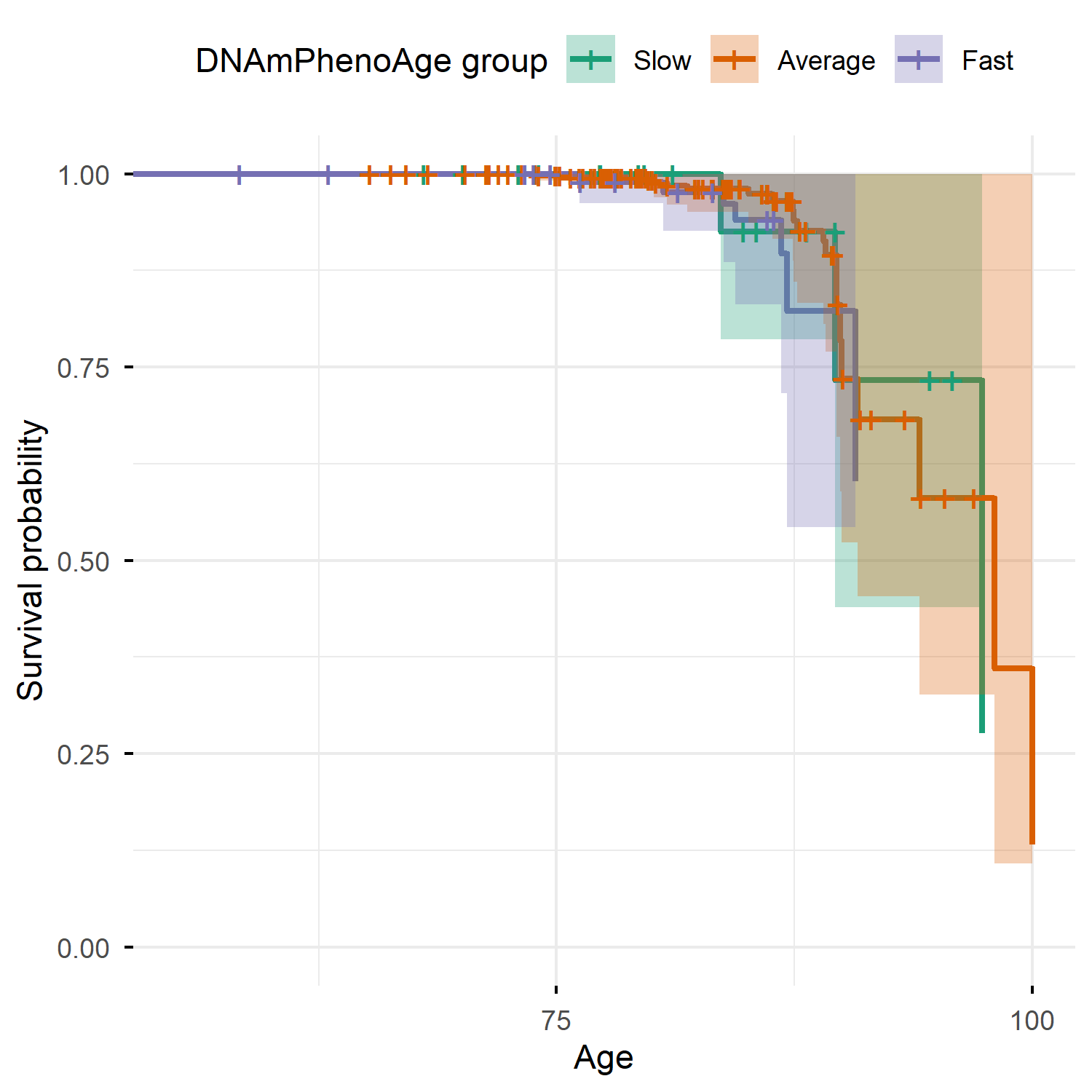 | B)  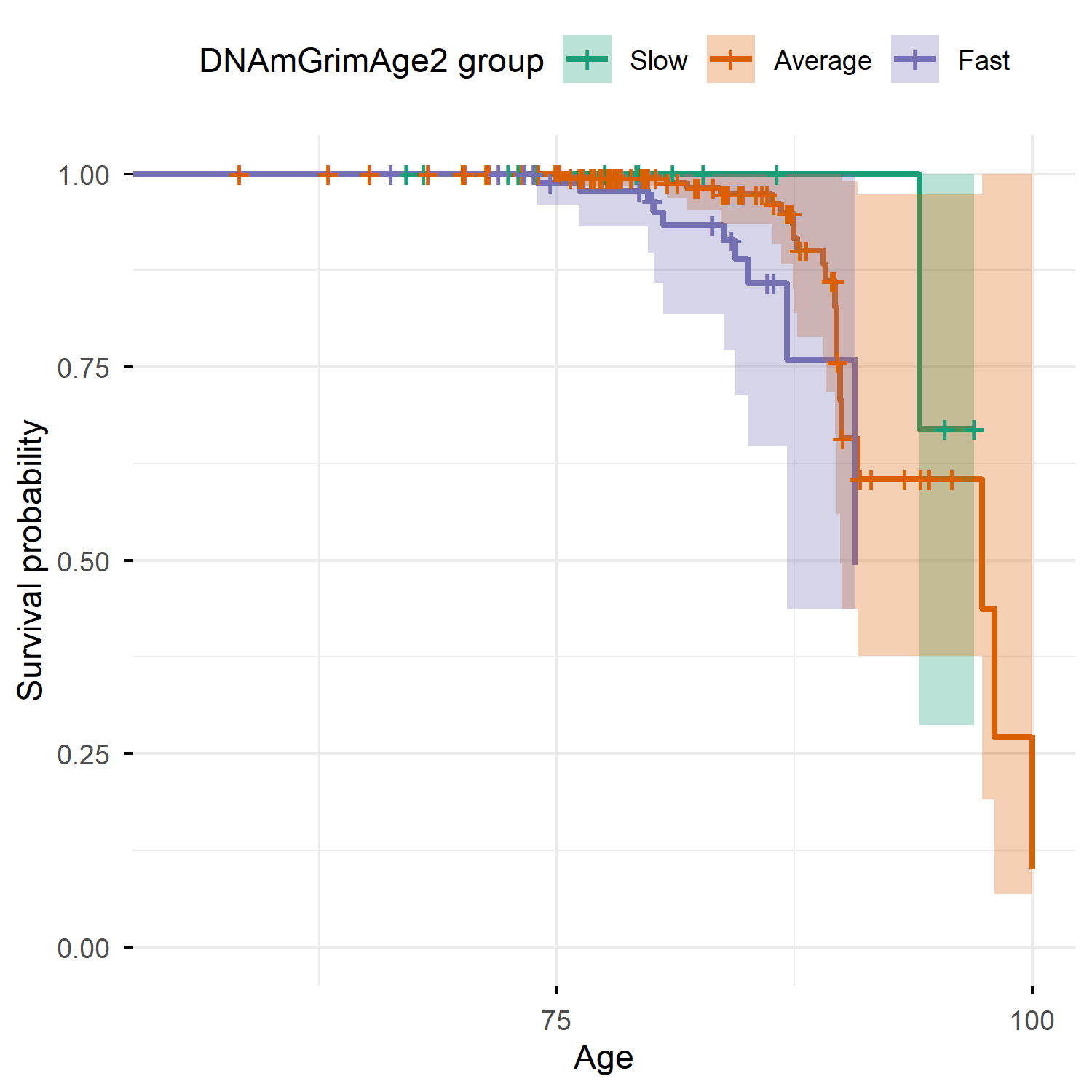 |
| --- | --- |
| C)  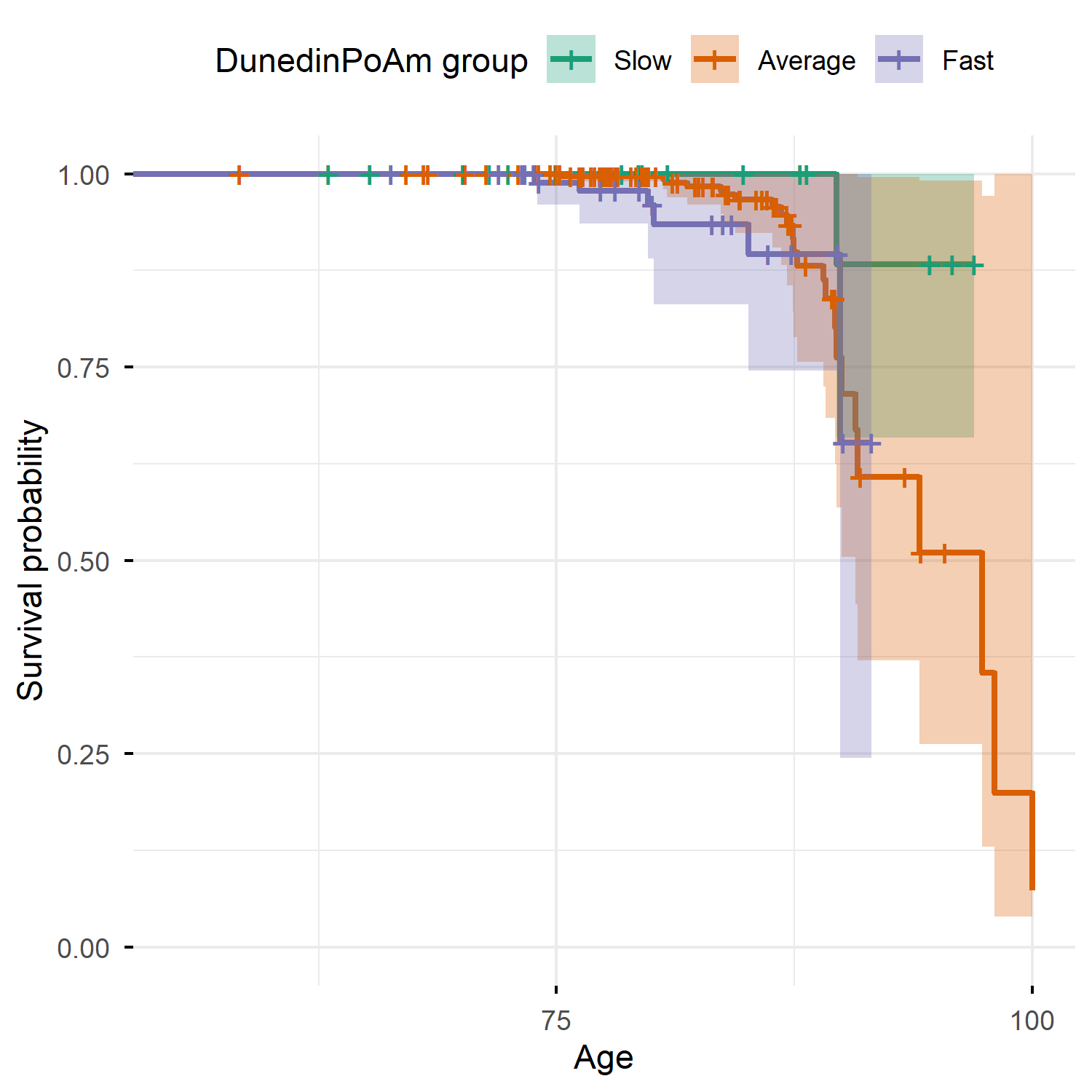 | D)  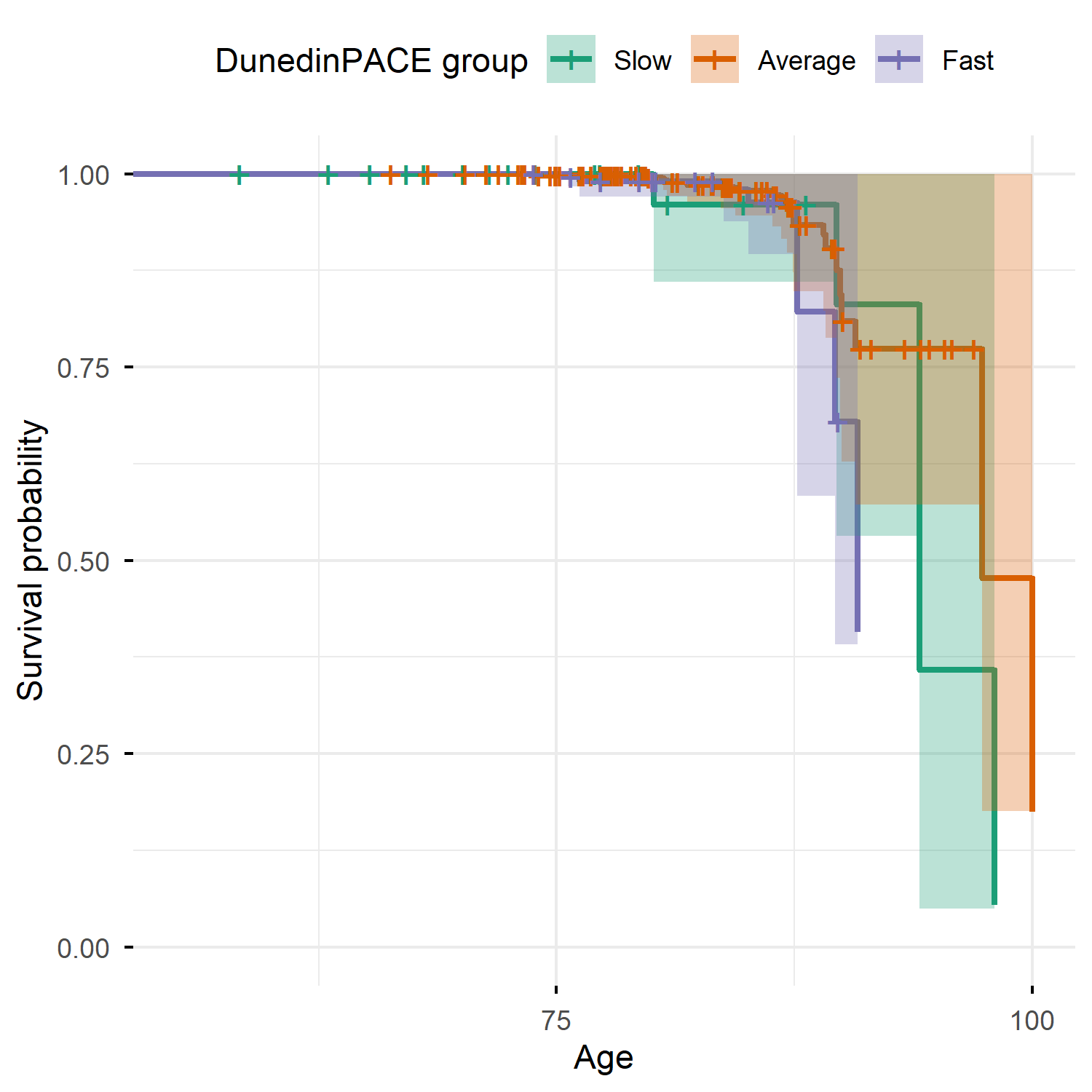 |

## References

1. Horvath S: **DNA methylation age of human tissues and cell types.** *Genome Biol* 2013, **14:**R115.

2. Hannum G, Guinney J, Zhao L, Zhang L, Hughes G, Sadda S, Klotzle B, Bibikova M, Fan JB, Gao Y, et al: **Genome-wide methylation profiles reveal quantitative views of human aging rates.** *Mol Cell* 2013, **49:**359-367.

3. Zhang Q, Vallerga CL, Walker RM, Lin T, Henders AK, Montgomery GW, He J, Fan D, Fowdar J, Kennedy M, et al: **Improved precision of epigenetic clock estimates across tissues and its implication for biological ageing.** *Genome Med* 2019, **11:**54.

4. Levine ME, Lu AT, Quach A, Chen BH, Assimes TL, Bandinelli S, Hou L, Baccarelli AA, Stewart JD, Li Y, et al: **An epigenetic biomarker of aging for lifespan and healthspan.** *Aging (Albany NY)* 2018, **10:**573-591.

5. Lu AT, Binder AM, Zhang J, Yan Q, Reiner AP, Cox SR, Corley J, Harris SE, Kuo PL, Moore AZ, et al: **DNA methylation GrimAge version 2.** *Aging (Albany NY)* 2022, **14:**9484-9549.

6. Belsky DW, Caspi A, Arseneault L, Baccarelli A, Corcoran DL, Gao X, Hannon E, Harrington HL, Rasmussen LJ, Houts R, et al: **Quantification of the pace of biological aging in humans through a blood test, the DunedinPoAm DNA methylation algorithm.** *Elife* 2020, **9**.

7. Belsky DW, Caspi A, Corcoran DL, Sugden K, Poulton R, Arseneault L, Baccarelli A, Chamarti K, Gao X, Hannon E, et al: **DunedinPACE, a DNA methylation biomarker of the pace of aging.** *Elife* 2022, **11**.
